# A bio-psycho-socio-planetary model of health and Iris Marion Young’s concept of responsibility as foundation for a medical ethos during environmental crises

**DOI:** 10.3205/zma001831

**Published:** 2026-03-23

**Authors:** Verina Wild

**Affiliations:** 1 University of Augsburg, Medical Faculty, Institute for Ethics and History of Health in Society (IEHHS), Augsburg, Germany

**Keywords:** health equity, environment, climate, social determinants of health, ethics, Professional Identity Formation

## Abstract

**Aim:**

In October 2022, the World Medical Association integrated environmental sustainability, public health, global health, considerations of inequities as well as the well-being of future generations into its revised *International Code of Medical Ethics* for physicians. Broader responsibility for environmental and societal issues was thereby codified into medical professionalism. Theoretical work on a normative foundation for such a comprehensive understanding of responsibility is scarce. This paper aims to contribute to the normative side of a contemporary professional identity formation (PIF) in medicine. It develops an “ethos in connection” which helps to justify broadening physician responsibility and integrating environmental and societal dimensions.

**Methods:**

It is asserted that defining and understanding *health* and *responsibility* are the decisive elements of the normative foundation for a contemporary PIF. The paper proposes a comprehensive bio-psycho-socio-planetary model of *health*. Relevant elements of the philosophical work by Iris Marion Young on *responsibility* are summarized. These comprise the social connection model, distribution of responsibility and relational egalitarianism.

**Results:**

The bio-psycho-socio-planetary model of health and Young’s theory of responsibility are integrated to establish an “ethos in connection” to provide the normative foundation guiding the PIF of today. Some challenging issues are discussed, for instance concerning whether and how it differs from a public health ethos and what practical implications follow.

**Conclusion:**

The paper concludes that the climate and environmental crises are not the decisive driving forces behind what this paper calls an “ethos in connection”, as the need for such an ethos has been prevalent long before these crises became issues of highest priority. Nonetheless, the severity of health impacts and the inequality in causation and distribution of the multi-layered climate and environmental crises increase the urgency to further develop the PIF of today.

## Zusammenfassung

**Ziel:**

Im Oktober 2022 integrierte der Weltärztebund ökologische Nachhaltigkeit, Public Health, globale Gesundheit, Überlegungen zu Ungerechtigkeiten sowie das Wohlergehen zukünftiger Generationen in seinen überarbeiteten *International Code of Medical Ethics*. Damit wurde eine umfassendere ärztliche Verantwortung auch für ökologische und gesellschaftliche Fragen verankert. Theoretische Arbeiten zu einer normativen Grundlage für eine solches Verständnis ärztlicher Verantwortung sind selten. Der Beitrag arbeitet die normative Dimension einer zeitgemäßen *Professional Identity Formation* (PIF) in der Medizin weiter aus. Es wird ein „Ethos der Verbundenheit“ entwickelt, das dazu beiträgt, die Integration von Umwelt- und Gesellschaftsdimensionen in die ärztliche Verantwortung und das ärztliche Ethos zu rechtfertigen.

**Methoden:**

Es wird argumentiert, dass Definition und Verständnis von *Gesundheit* und *Verantwortung* die entscheidenden Elemente der normativen Grundlage für zeitgemäße PIF sind. Dieser Beitrag schlägt ein umfassendes bio-psycho-sozio-planetares Modell von *Gesundheit* vor. Außerdem werden relevante Elemente der philosophischen Arbeiten von Iris Marion Young über *Verantwortung* eingebracht: soziales Verbindungsmodell, Verteilung von Verantwortung und relationaler Egalitarismus.

**Ergebnisse:**

Das bio-psycho-sozio-planetare Modell der Gesundheit und Youngs Theorie der Verantwortung werden miteinander integriert, um ein „Ethos der Verbundenheit“ zu entwickeln, das die normative Grundlage für PIF von heute bildet. Es werden einige Fragen erörtert, z. B. der Unterschied zu einem Ethos im Bereich von Public Health und die sich daraus ergebenden praktischen Auswirkungen.

**Zusammenfassung:**

Es wird argumentiert, dass die Klima- und Umweltkrisen nicht die entscheidenden Triebkräfte für das hier beschriebene „Ethos der Verbundenheit“ sind. Die Notwendigkeit dafür bestand schon lange vorher – bereits bevor diese Krisen zu Themen höchster Priorität wurden. Allerdings erhöhen die Schwere der gesundheitlichen Auswirkungen der vielschichtigen Klima- und Umweltkrisen und die Ungleichheit ihrer Verursachung und Verteilung die Dringlichkeit, PIF von heute weiter zu entwickeln.

## 1. Introduction

It has been argued for many years that medical professionals have social responsibilities beyond the individual patient, e.g. in relation to social determinants of health or economic pressures on health care delivery [1], [2], [3], [4], [5], [6], [7]. Recently, given the ubiquitous and unequal health consequences of the environmental and climate crises, discussions about physicians’ roles and responsibilities are increasing [8], [9], [10], [11], [12], [13]. A “Planetary Health Pledge for Health Professionals in the Anthropocene” published in the *Lancet* proposed an interdisciplinary pledge based on the Declaration of Geneva (DoG) and called for a commitment within health professionals, including physicians, to work for planetary health and transformative change [12].

While the nursing profession has been outspoken about its environmental and social responsibilities for decades [14], parts of the medical profession have been critical about or even opposed to any broadening of physician responsibilities beyond the individual patient. A recent paper responded critically to the *Lancet* pledge, arguing that it would be “dangerous” to change the professional ethos as laid out in the DoG. Instead, the paper argued that the focus on the individual patient is and should remain the core professional responsibility, and that changes “are critical for the patient–physician-relationship and the trust in medicine” [15]. With regard to including social responsibilities in medical education and professional identity formation (PIF) it has been claimed “[m]aking social justice a distinctively professional imperative is a category mistake” and “the virtue of social justice is far beyond the power of medical education to produce. Any attempt to do so will certainly fail” [16]. 

At first sight, this separation aligns with conceptual distinctions between patient-oriented biomedical ethics that focuses on the *individual* and public health ethics which focuses on the *population* level [17].

And yet, in 2022, the WMA International Code of Medical Ethics (ICoME) has included environmental sustainability, public health, global health, considerations of inequities as well as the well-being of future generations into its revised code (see table 1 (Tab. 1), and [18], [19]). The revised Declaration of Helsinki, as influential research ethics code, also includes a passage on the requirement for medical research to be environmentally sustainable, and the need to consider contexts of structural inequalities and disparities is mentioned [20]. 

These developments are the paper’s starting point. As part of this special issue its aim is to contribute to ongoing developments in the field of PIF. PIF in medicine captures how physicians develop, think, act and feel as professionals in their individual, relational and collective identity [21]. The paper is based on the premise that PIF has both an empirical, descriptive side, describing how physicians grow into and perform their role; and also a distinctively normative, prescriptive side raising claims which role medical professionals should grow into and how they *should* perform it. This paper seeks to deepen the normative dimension and to ground it in a defined ethics.

Existing normative sources in bioethics often ground medical professionalism and ethos in the four principles of biomedical ethics [22]: autonomy, beneficence, nonmaleficence and justice. Despite attempts to include care for climate and environment [23], principlism with its emphasis on personal autonomy has been criticized for its overly individualized perspective [24].

Gils-Schmidt and Salloch recently developed a new approach and conceptually grounded physician ethos in Korsgaard’s neo-Kantian moral framework, showing how the moral duty to protect the climate can and should follow from a physician’s identity of being a healthcare professional [25]. While their paper provides a strong ethical justification for expanding professional responsibilities to include care for the climate, there is altogether very little theory-based work on the normative foundation of such a more comprehensive understanding. By developing an “ethos in connection”^1^, this paper aims to help filling this gap.

As the paper will show, insights inspired by Iris Marion Young’s philosophical work will help to discharge potential tensions between the individual and the social/environmental level [26], [27], [28]. I will argue that it is conceptually false to conceive the individual versus the societal and/or environmental levels as conflicting poles, because they are so deeply interconnected, best captured in a bio-psycho-socio-planetary model of health. Instead of confrontation, integration is the goal. I will assert that the climate and environmental crises are not the decisive driving forces for the need of an “ethos in connection”, as it was necessary long before climate and environment became issues of highest priority in health. But the highly unequal health impact of the multi-layered climate and environmental crises increases the urgency to incorporate these obligations into the PIF of today.

## 2. Health and responsibility as core elements of an “ethos in connection”

It is undisputed that physicians are responsible for their patients’ health and wellbeing. For centuries this has been the defining normative core of physicians’ professional identity. The WMA Declaration of Geneva says: “The health and well-being of my patient will be my first consideration” [20], and the above cited ICoME states: “The primary duty of the physician is to promote the health and well-being of individual patients by providing competent, timely, and compassionate care in accordance with good medical practice and professionalism” [19]. 

From a normative point of view the interest lies in the words *health* and *responsibility*. The definition and understanding of these words provide the decisive core of the normative foundation for a contemporary PIF. We will first turn to health. 

### 2.1. Health

Defining and understanding *health* has a long tradition in empirical and normative scholarship [29], [30]. This paper supports a bio-psycho-social model of health [31], [32], [33] while acknowledging that it is not universally shared. Many implicitly or explicitly teach and practice the biomedical model of health, focusing on biological processes in individual human beings [34]. I nevertheless adhere to the more comprehensive bio-psycho-social model, adding, as I will explain below, a planetary dimension, resulting in a bio-psycho-socio-planetary model of health.

For decades, vast amounts of empirical data show the effect not only of biological and psychological but also social determinants on health and disease [35]. The unequal distribution of these health determinants leads to a social gradient in health for almost all diseases: socio-economic positions correlate with mortality and morbidity [36]. Structural racism and stigmatization within health care systems further drive health inequalities [37], [38]. These findings have motivated a large body of literature on health justice and have shaped the understanding of health and disease as being inextricably connected with its social conditions [30], [39], [40], [41], [42].

While social determinants are mostly understood to include environmental aspects [43], I want to explicitly emphasize the planetary environment. Health as connected to its planetary environment has been captured for millenia in Indigenous knowledges and scholarship and in more recent approaches such as one health, eco health and planetary health [44]. A dynamic debate is ongoing whether such integrative approaches are distinct, how they relate to social determinants of health, and what is meant by “planetary” [45], [46], [47]. Without wanting to get into too detailed discussions about terms and boundaries, I focus on what can be learned from these approaches. All of them confirm the interconnectedness and interdependency of human health and disease with the physical and planetary environment. By adding the word *planetary* to the bio-psycho-social model, I seek to explicitly incorporate the non-human environment, consisting of e.g. the climate, air, vectors, animals, plants, fungi and water. 

An illustrative example for the planetary interconnectedness of health is antimicrobial resistance (AMR). Prescription and use of antibiotics for humans and animals in health care and livestock husbandry, as well as its high levels in wastewater, are directly related to the reduction of antibiotic effectiveness, which is becoming a significant problem for medicine today and in the future [48]. 

Interrelations between health and anthropogenic climate change are also well established. Climate change negatively affects human health for example through unequally distributed effects of heat, vector-related diseases, mental health burdens, famines or displacement, while the health care sector itself significantly contributes to climate-harming emissions [49]. 

It follows that any comprehensive understanding of human health can never be disconnected from its social and planetary determinants. 

### 2.2. Responsibility 

The understanding of *responsibility* that underpins the “ethos in connection” developed in this paper is based on work by the philosopher Iris M. Young. As we have seen above, many academic contributions demand teaching of social responsibility in the education of medical students [50], [51] so far without recurring to Young’s work. Instead, Young’s work has already been fruitfully applied to the domain of population-based and global health [52], [53], [54]. Furthermore, Young’s work has been used to convincingly ground an individual ethos concerning global structural injustices [28], [55]. While Young’s work addresses all individuals, three elements are particularly relevant for the professional medical ethos in my opinion:

1. Social connection model [26]
2. Distribution of responsibility [26]
3. Relational egalitarianism [27]

Since they are still new to the field of medical ethics and PIF, they will be broadly summarized. Section 3 will adapt and apply them to medical ethos and PIF.

1. *Social connection model*: In her social connection model, Young analyses how agents should think about responsibility in relation to structural injustices [26]. She affirms that in relation to complex structural injustices, a linear connection of responsibility cannot be traced from one individual to the harm that is being done to another individual and that in this way harm to others is often unintended. The model she develops shows how all those who “contribute by their actions to structural processes with some unjust outcomes share responsibility for the injustice” ([26], p. 96). The responsibility is not backward-looking, but forward-looking: it is not about assigning blame, but rather as determining that an individual should join others to transform structural processes to make outcomes less unjust. Young writes that “our responsibility derives from belonging together with others in a system of interdependent processes […]. Responsibility in relation to injustice thus derives not from living under a common constitution, but rather from participating in the diverse institutional processes that produce structural injustice.” ([26], p. 105). In her much-cited paper on responsibility for addressing the harms occurring in the context of the global apparel industry and the social movement seeking changes in its exploitative working conditions, Young substantiates her model of responsibility and shows that it is not about individual culpability but about taking forward-looking, shared political responsibility for addressing and overcoming harms done within structures and processes [56].
2. *Distribution of responsibility: *According to Young, all agents that, in one way or another, are “socially connected” to the structural injustices share responsibility for remedying them. It is “up to the agents who have responsibility to decide what to do to discharge it within the limits of other moral considerations.” ([26], p.143). Agents have a right and a responsibility to criticize others with whom they share responsibility for not taking enough action, taking ineffective or counterproductive action. To provide some guidance on how to reason about taking action, Young develops four parameters of reasoning. She suggests that individuals should think about their position of power: individuals should focus on those harms where they have greater capacity to influence structural processes, such as institutions, social norms and practices. Privilege is the second parameter to think about: persons with greater privilege have greater responsibility to take action. Interest is the third: responsibility derives from a shared interest in reducing harm. Lastly, agents should think about their collective ability, as “some agents are in positions where they can draw on the resources of already organized entities and use them in new ways for trying to promote change” ([26], p.147).
3. *Relational egalitarianism: *Justice, in general, can be defined in distributive terms (e.g. who gets what and how much?) and in relational terms (e.g. how do people relate to one another?). At the core of Young’s work is the conviction that injustices should be defined primarily in terms of relations: instances of oppression and discrimination need to be identified and remedied [27]. She is hence a proponent of relational egalitarianism, a theory which has proven to be fruitful in discussions of health justice, public health and individual responsibility for health [57], [58], [59], [60], but not yet in relation to medical professionalism. For the following section we will keep in mind that justice should be conceived of as relational and that power differences and the processes of oppression and discrimination matter in relations.

## 3. The “ethos in connection” as a theoretical foundation for Professional Identity Formation

The elements described in section 2 will now be combined to develop what I call an “ethos in connection” as a normative foundation for PIF. 

Young centers her work around structural injustices for which responsibility should be taken. We will now focus on health and health injustices, for which responsibility should be taken. While Young’s theory addresses every moral agent and any collective, her work can be adapted to focus on medical professionals as individuals and as a collective. 

Young’s work on social connections and structural injustices affirms that the individual cannot be considered as isolated or disconnected from their surroundings. Just as Young resists separating the individual and the structural, seeing them as connected, so the connections between individual bio-psychological and broader socio-planetary dimensions should be affirmed in the medical field. As we have seen in section 2.1., all dimensions of health are inextricably interconnected. This, then, should be the core responsibility for the medical profession: *when caring for someone’s health, it means caring for someone’s bio-psycho-socio-planetary health*. For example, a practical intervention on the individual level, such as prescribing an antibiotic or using a climate-damaging drug, needs to be considered including its contribution to AMR or its climate impact. 

Young’s work explains that for many harms no single individual can be blamed. In our case, we can apply this, for example, to the negative health impact of climate change, to poverty-related health inequalities or to structural racism in health care. Young’s model of responsibility demonstrates that individual physicians cannot be blamed in linear connection, and yet some form of professional responsibility exists. By assigning responsibility in a forward-looking way, it is often neither possible nor necessary to identify any malevolent intent to cause harm. Hence, the knowledge about the inextricable bio-psycho-socio-planetary connections oblige all medical professionals in their PIF to take responsibility accordingly. 

To clarify physician responsibility, PIF can make use of Young’s four parameters of reasoning. Medical professionals are connected in their collective *interest* in health. They are a highly respected group with the *privilege* of access to expert knowledge and with epistemic and social authority to process, use and distribute it as well as significant *power* to influence even political decision-making. Through professional associations on regional, national and international levels they have strong *collective abilities*. This establishes and emphasizes their responsibility to care for health and to take action against health harms, including unequally harmful biological, psychological, social and planetary determinants of health. It is thus a matter of their professional responsibility, and not of private political convictions, to proactively address any processes that impact and potentially harm health, including e.g. poverty, climate change or racism. 

Such acts must not and should not be seen to be in tension with responsibilities to support the health of individual patients but as ultimately strengthening it. While caring for the individual patient, all actions that can be *reasonably *performed to improve health and minimize harm, should be performed. How far these reasonable actions should go depends on the situational context. It is a matter of further discussion and will partly be addressed below. At least it should be clear that responsibility for health cannot be conceptualized on an isolated individualized level.

Young argues that justice should primarily be concerned with relations, in particular the quality of interactions among humans and more specifically with reducing oppression and discrimination. The principle of justice figures prominently in teaching medical ethics and the formation of a medical ethos, but it is usually conceptualized as distributive justice. Expanding the understanding of justice beyond the distributive dimension to include, even prioritize its relational dimension, emphasizes the imperative to address oppressive and discriminatory relations in the medical context, particularly in the patient-physician relationship [61]. Young provides inspiring ideas on participation and inclusion that can provide a theoretical foundation for already existing activities in medical practice and education [62], [63]. Again, assigning responsibility is not about blaming individuals for harmful behaviour but rather about understanding that domination and oppression are structural and should be countered by all who are connected to it. This means that in their PIF every medical professional has an obligation to learn about structural oppression and to think about how to take action against it, either in the individual patient encounter or on the level of teaching, research or as agents of professional associations. 

Taken together, the bio-psycho-socio-planetary model of health and Young’s theory of responsibility help to establish an “ethos in connection” that can, and, as argued above, should form the normative foundation guiding PIF of today. 

## 4. Discussing an “ethos in connection”

Is a physician’s “ethos in connection” different from a public health professional’s? The comprehensive conceptualization of health and responsibility and the overall interest in advancing health seems similar for both professions. But the professions occupy distinct positions within the domains of health and health care and as such have different powers, privileges and collective abilities resulting in distinct role-related specific responsibilities and actions. 

The first principle of established medical codes shall not be questioned here, that is, that physicians are responsible for the health and wellbeing of their patients. On the contrary, this imperative shall be strengthened by including some form of responsibility for all relevant dimensions. Medicine and public health differ in the content of teaching and in ways of expressing their ethos through decisions and actions. Medical professionals need to learn about all dimensions of health and treatment, including the social and planetary dimensions, but undoubtedly also including the individual, even microscopical dimensions, pathophysiology or pharmacology. The latter are contents that public health students do not necessarily have to learn in such detail. For a medical professional it is also of central importance to learn how to build a professional relationship with the individual patient. Not everyone who designs public health interventions must be taught in building relations with individual patients.

In light of this, one may wonder: if the person standing in relation to the medical professional is the primary unit of concern, doesn’t the “ethos in connection” then collapse into a theoretical concept without much impact on practical implications? On the contrary. The “ethos in connection” has significant practical implications for teaching and practicing medicine. 

Imagine a person coming into a medical practice with elevated blood pressure. After medical history taking, it is clear that climate effects and experiences of poverty, discrimination and violence are co-causing the blood pressure to rise. While the physician can prescribe a drug to reduce the blood pressure, they cannot *directly* change the circumstances of climate change, poverty and violence the patient is exposed to. Some may think that physicians do not have and should not have any responsibility for the broader issues and the background of a person's health. Instead, it should be public health officials or political work.

According to the concept of the “ethos in connection” this view should be considered as overly narrow and misguided, even harmful. It repeats the false dichotomy that pits the individual against the socio-planetary. To comprehensively understand the individual pathophysiology and treatment of a disease means to be aware of the complex interconnections of the bio-psycho-socio-planetary dimensions. Even if climate change or poverty cannot be immediately changed, taking responsibility includes professional obligations to learn about health comprehensively, to research and to teach it. The data about the effect of social and planetary determinants on health should be known, just like the individual effects of genetic determinants on health; and medical curricula and conferences should feature these topics. This can lead to a better understanding of the patient’s individual situation, a better acknowledgment of the limits of a biomedical approach [64], [65] and to constructive thinking about potential collective activities as a profession. 

Power, privilege and collective ability of medical professionals increase the responsibility to become active within respected professional associations collectively, as agents and advocates, and to address harms to health due to socio-planetary determinants, such as climate change, poverty and racism. Medical professionals should also find out or be taught how structural oppression and discrimination within the medical system can influence their own conscious and unconscious biases and behaviours and what can be done to manage them better in their professional relations with patients. 

Some might still object to the proposed “ethos in connection” arguing that it should not be the individual physician’s responsibility to decide e.g. whether to withhold a climate-harming drug from a patient to protect public and global health. This paper does not claim that such decisions should always and only be made by individual physicians. Its main argument is compatible with views that such decisions should be made responsibly and collectively on a higher level. However, if this objection is made, this, again, might fall into the trap of polarized thinking. The “ethos in connection” means that medical professionals should always seek to understand themselves as responsible agents within structures, and seek to understand and where possible address, all determinants of their patient’s health. This extends to obligations that bind the profession to act collectively if risks and harms are detected. It can also involve prescribing the drug or performing the intervention for the sake of the individual patient, but not without further thinking. This is well-captured, for example, in the concept of antibiotic stewardship, which calls for physicians to use antibiotics rationally and responsibly, in full knowledge of the potentially harmful consequences [66]. Responsibility can and should also be addressed by working through professional associations and beyond, to press for collective and political action. 

Grounding professional identity and its development in an “ethos in connection” helps to open spaces for thinking about, teaching and practicing medicine in a constructive and health-centered way, instead of constructing barriers to improving health because “broader” aspects are wrongly perceived not to be within the scope of medical professionals. 

## 5. Conclusion

This paper proposes that a bio-psycho-socio-planetary model of health and the work of Iris M. Young can strengthen the normative side of professional identity formation in medicine. The aim is to further encourage ethics and ethos-building for physician responsibility to include attention to social and planetary determinants of health.

This paper cannot offer more than a first sketch and opens many avenues for future work, such as a more systematic description of the connection between the individual and their environment; a deeper analysis of public health ethics in contrast or in congruence with medical ethics; more work on concrete action in challenging test cases; details on how to teach this ethos; and analyses as to whether this approach can be integrated into principlism or whether it must stand as an alternative account. 

Climate change and its unequal impact on health should be considered in PIF, but it is not decisive in conceptualizing the “ethos in connection”. As mentioned, a broader understanding of physician responsibility has been discussed for many years, also without connection to climate change. Nonetheless, given the scale of the threat to human health and health equity from anthropogenic climate change, there is a need to act now. This increases the urgency to think about contemporary medical ethos and professionalism. And yet, the urgency should not lead to a myopic or sole focus on climate and environmental crises. This paper argues in a broader and more foundational sense. 

Concerning this, much is already under way within the medical profession. It is increasingly argued for the need to understand and acknowledge dimensions of professional responsibility that situate the presenting patient within their broader social and environmental context. This paper seeks to provide normative support for integrating professional obligations to incorporate the bio-psycho-socio-planetary dimensions of health. No physician and no teacher at a medical faculty should have to defend themselves for doing this, nor should there be a basis for criticizing them for doing so. On the contrary, it would be the core of their professional duties to act in responsibility for health. 

## Acknowledgements

Many thanks to Jan-Christoph Heilinger, Julian Sheather, Cristian Timmermann, Katharina Wabnitz, Alistair Wardrope for reading versions of this article and for helpful comments, and to Michael Köberle, Fabian Linder, and Isabel Fernandez Mayor for editorial support. Many thanks to Ryoa Chung, James Dwyer, Lisa Eckenwiler, Carina Fouria, Agomoni Ganguli Mitra, Jan-Christoph Heilinger for years of reading and discussing the work of Iris M. Young and discussing this work in relation to health.

## Note

^1^ By adding “in connection” I am not intending to develop a specialized ethos, applicable only for some. Ultimately, I argue that the medical ethos should always be understood in this way and therefore should not require any further addition.

## Funding

This work is partially supported by a Seed Funding Grant on “Environmental Health Ethics and Justice” from the University of Augsburg. 

## Author’s ORCID

Verina Wild: [0000-0003-3012-7662]

## Competing interests

The author declares that she has no competing interests.

## Figures and Tables

**Table 1:**
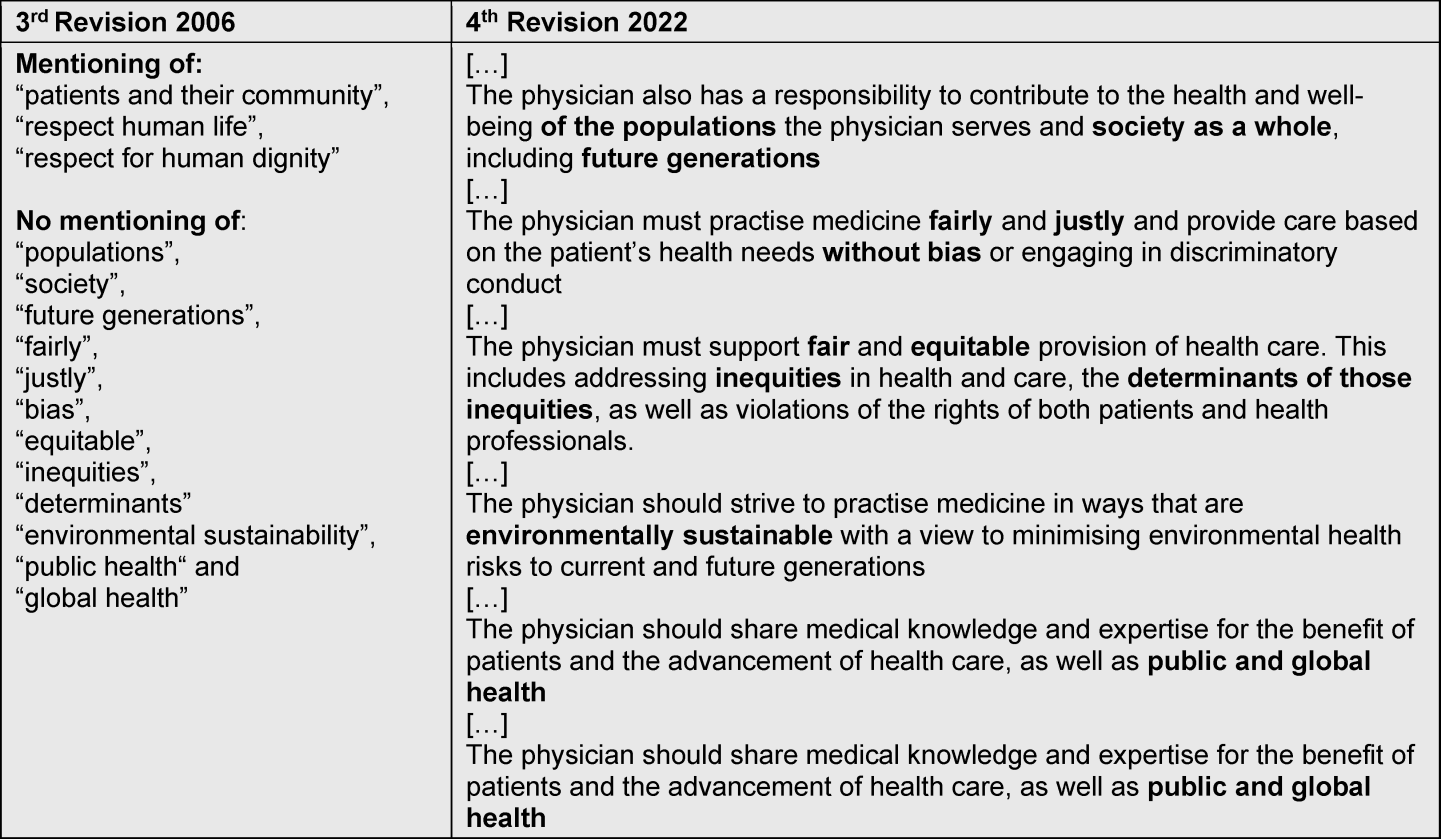
Comparison of parts of the 3^rd^ (2006) and 4^th^ (2022) version of the WMA International Code of Medical Ethics
